# Miscellany

**Published:** 1903-07

**Authors:** 


### Miscellany.

Chipping of Porcelain Teeth in Soldering.—In a communication to the *Dental Cosmos,* March, 1903, Dr. O. E. Wall, of Honolulu, recommends coating the porcelain facings with a rather thick coat of ordinary sperm oil after they are ground and backed, and before waxing up, to prevent chipping at the incisal and approximal edges. After waxing up he recoats the facings freely with oil and invests. When the wax is removed he applies oil to the investment wherever borax is likely to penetrate, using for this purpose a camel's-hair brush. He was led to do this by the impression that the chipping was due to borax coming in contact with these portions of the teeth. This the oil effectually prevents, and he writes that since adopting this procedure his failures from teeth chipping at the edges have been lessened fully ninety per cent.—W. H. T.

---

Celluloid Dentures.—In continental Europe a large number of celluloid dentures are made every year and are giving general satisfaction. Proper methods are employed in their construction and better material is used than that sold on this side of the ocean. The "French Company of Celluloid," of Paris, makes a celluloid which is far superior to that sold by our dental supply houses, but unfortunately that firm has not yet offered it for sale to the dental profession. Through indirect sources I have obtained a small quantity of it in the shape of sticks, and, with the aid of a series of dies and counterdies of different sizes, blanks have been made. These blanks I have put aside in a dry place to season. Their exposure to sunlight has caused them to fade slightly, and this gives them a still more natural appearance. It is my intention to give my blanks some few years to season, and when ready to use them I expect to have a material far superior to any ever placed on the market.

Very little has to be said about repairing celluloid dentures, as they rarely need repairs, excepting the replacing of a tooth. This is done very much as with a rubber denture. After removing the wax the filed portion should be rubbed with acetone, a small piece of celluloid placed in position, and the case pressed as if moulding a plate. For partial dentures the process is identical, with the exception that the blank is trimmed to suit the case in hand.—Dr. Louis F. Bouche, Winnipeg, Manitoba, Canada.

---

Orthoform Ointment.—Dr. A. D. Kyner, in *Items of Interest,* reports success in use of orthoform ointment, twenty-five per cent. in cases where arsenic had been in contact with gum.

